# Perspective: Food and Nutrition Insecurity in Europe: Challenges and Opportunities for Dietitians

**DOI:** 10.1016/j.advnut.2023.07.008

**Published:** 2023-08-03

**Authors:** Elena Carrillo-Álvarez

**Affiliations:** Public Health Specialist Network (ESDN PH), European Federation of Association of Dietetics (EFAD), Europe; Global Research on Wellbeing (GRoW) research group, Blanquerna School of Health Sciences, Universitat Ramon Lull, Barcelona, Spain

**Keywords:** food insecurity, food and nutrition insecurity, Europe, dietitians, nutritionists, health disparities

## Abstract

In recent years, the interest in food and nutrition insecurity in high-income countries has skyrocketed. However, its recognition in Europe is still developing. This perspective summarizes the evidence on food and nutrition insecurity across Europe in terms of prevalence, consequences, and current mitigation strategies, with the aim of outlining the challenges and opportunities for dietitians. Prevalence in the general population ranges between 5% and 20%, with higher rates identified in women, children, older adults, single-parent households, those with low educational attainment, and on low or unstable income and/or employment. In users of food aid, the prevalence of food insecurity is above 70%. Responses to food and nutrition insecurity include welfare policies and food assistance programs at regional and national levels. However, most current strategies are not successful in tackling the structural drivers of food and nutrition insecurity, nor do they guarantee diet quality. Despite limited involvement to-date, dietitians can play an important role in addressing food and nutrition insecurity across Europe. This narrative identifies 4 areas: *1*) create awareness of the existence and severity of food and nutrition insecurity, *2*) advocate for comprehensive, robust data on the determinants and prevalence, *3*) partner with diverse stakeholders, social assistance providers, local authorities, and nongovernmental organizations in a comprehensive, intersectoral, and integrated manner, *4*) participate in the development of political instruments and interventions that ensure equitable access to high-quality safe food.

**Statement of Significance**

Food and nutrition insecurity is an underrecognized problem in Europe. This perspective article delves into its prevalence, associations, consequences, and existing measures, aiming to highlight the significance of dietitians’ involvement in ensuring sufficient food and nutrition for everyone.

## Introduction

In recent years, interest in food and nutrition insecurity in high-income countries has skyrocketed, as a result of initially the 2008 economic crisis [[1], [2], [3]], then the COVID pandemic [[4], [5], [6]], and more recently the Ukrainian conflict and the rise in global inflation [7,8]. Food insecurity entails not having physical, social, and economic access to adequate, nutritious, and safe food that satisfies dietary needs and food preferences [9,10]. Nutrition security is achieved when dietary and nutritional needs are satisfied [9]. In this perspective piece, the term food and nutrition insecurity emphasizes that both entities must be recognized, as nutritional sufficiency can only be achieved through a stable provision of healthy foods, and that the lack of access to adequate food has an independent impact on health and well-being.

Food and nutrition insecurity is a worldwide concern due to the physical and psychological burden it imposes. It reduces dietary diversity and micronutrient deficiencies, particularly iron, zinc, and vitamin B [[11], [12], [13]], across the lifespan. Low household food and nutrition security impacts infants and children, leading to an elevated risk of low birth weight, infant anemia, and compromised immunity [14,15]. Moreover, it is associated with impaired physical and psychological development, including poor academic achievement, emotional, behavioral, and relational outcomes that can persist into adulthood [14,15]. It is associated with excessive energy, carbohydrates, saturated fat, sugar, and salt consumption in children, adolescents, and adults living in high-income countries [14,16]. Food-insecure older adults consume less energy and macronutrients, have less muscle mass and higher rates of chronic disease, and report poorer health than their food-secure counterparts [17,18]. Food and nutrition insecurity in high-income countries increases the likelihood of cardiometabolic risk factors, increases the incidence of diabetes, hypercholesterolemia, and hypertension. Moreover, it exacerbates other chronic conditions, including asthma and poor oral health [3,[16], [17], [18], [19], [20]]. It also increases the likelihood of adolescents and adults gaining excess weight [21,22]. Apart from these consequences, a bidirectional relationship exists between food and nutrition insecurity and mental health issues [[23], [24], [25]], as well as negative repercussions for work performance and healthcare expenditure [[26], [27], [28]].

Research on food and nutrition insecurity has a relatively established tradition in high-income countries like the United States, Canada, Australia, and the United Kingdom, where prevalence rates are between 4% and 12% [29,30]. However, research is only just emerging in Europe, despite almost 8% (∼58 million people equivalent to the Italian population) of the European population experiencing moderate or severe food insecurity^1^ [31].

Food and nutrition insecurity constitutes a complex global challenge involving a multitude of system actors, sectors, and stakeholders [[31], [32], [33]]. This article seeks to examine the potential role of dietitians in achieving food and nutrition security in Europe. To accomplish this, this study outlines the components of household food and nutrition insecurity in Europe, how it is measured, and the current mitigation strategies. Consequently, this perspective delineates the challenges and new directions for dietitians to facilitate their contribution to the fight against food and nutrition insecurity in Europe.

### The conceptualization of food and nutrition insecurity

The terms “food security,” “nutrition security,” “food and nutrition security,” and “food security and nutrition” are interrelated concepts frequently used interchangeably, despite conceptual differences.

The FAO defines food security as the condition in which “all people have physical, economic, and social access to sufficient, safe, and nutritious food to maintain a healthy and active life at all times” [34]. This definition used to encompass 4 dimensions, availability, access, utilization, and stability [35]. However, it has recently been expanded to include agency and sustainability [31,36,37]. “Availability” indicates that food is readily accessible to citizens. It depends on food production, stock levels, and net trade, and is generally analyzed at the country level. In high-income countries, the focus is not necessarily on the quantity of available food, but rather on the availability of nutritious food and the maintenance of supplies despite natural disasters and civil conflicts. “Access” refers to how individuals reach the available food in their environments, and it has 3 essential components that can be evaluated at the household or individual level: physical (for example, transportation), economic (for example, prices, cost of living, and incomes), and social (for example, social wages). This aspect of food insecurity has received the most research [[38], [39], [40], [41]]. Most monitoring tools for food insecurity evaluate economic access [42,43]. Meanwhile, “utilization” has 2 aspects [44]. The first is that individuals can consume food to ensure proper nutrition. This aspect is essential for food safety, adequate sanitation, and safe water. The second aspect relates to the conversion of food into meals and includes elements like intra-household distribution, purchase and conservation practices, food preparation, food literacy, and equipment for food preparation and storage. “Agency” describes the capacity of individuals or groups to make their own decisions regarding the foods they consume; how those foods are produced, processed, and distributed within food systems; and the opportunities for citizens to participate in processes that shape food-system policies and governance. As such, it is closely related to “food sovereignty” [45]*.* Meanwhile, “sustainability” is the long-term capacity of food systems to provide food and nutrition security without compromising the economic, social, and environmental systems on which food production and consumption depend for future generations.

One of the strengths of this definition is the recognition that food security involves ensuring not only the right combination of foods to provide the necessary nutrients for health, but also the production, procurement, cooking, eating, and sharing of food in a socially and culturally acceptable manner. In this regard, the term “food and nutrition security” has been suggested as a more accurate representation of the concept [46], with some authors arguing that “nutrition security” should suffice [47,48]. FAO continues to use the terms “food security and nutrition” globally [49]. A further development in the conceptualization of food security is its framing as the human right to adequate food [36,50,51]. From a socioecological perspective, food (and nutrition) insecurity is primarily a social problem with nutritional consequences. According to Huberland et al. [52], food insecurity is the “inadequacy between necessary and available resources” along” 2 dimensions: at the access level, for financial, temporal, informational, and freedom of action; and at the food use level, for temporal factors, material, knowledge, and skills.” In this way, food insecurity in Europe threatens not only the biological function of food (that is, nutrition), but also significant social and cultural roles, such as sharing with loved ones, celebrating, and savoring special foods (for example, home-cooked desserts and family recipes). Ultimately, food insecurity impedes the ability to consume an adequate and health-promoting diet and choose what to eat, how to acquire, prepare, and consume food, thus compelling individuals and families to satisfy this fundamental need in socially suboptimal ways.

### Food and nutrition insecurity measurement in Europe

The measurement of food insecurity in high-income countries has been elaborately described elsewhere [[38], [39], [40],[53], [54], [55], [56]]. Various classifications of such approaches include nation-based estimates of food insecurity, scales to assess household food access and acquisition, analyses of food affordability, and measures of dietary diversity and undernutrition. For this perspective piece, a summary of those utilized in the European region is presented to provide an overview of the available measures and contextualize the data presented in the following sections.

Most European countries do not regularly monitor national food insecurity [57]. For example, Portugal and the United Kingdom are exceptions to this pattern [58,59]. The food-related material deprivation indicator in the EUropean Survey on Living Conditions (EUSiLC)—“cannot afford a meal with meat, fish, or vegetarian equivalent every second day”—is the most closely related aspect of food and nutrition insecurity that the European Union routinely monitors [60]. This indicator is conceived to reflect an inability to access certain foods that are central to a healthy diet. However, it offers limited information about other essential food groups, the complex phenomenon of food and nutrition insecurity, and the relationship between an individual’s income and ability to access food [61]. There are 2 main reasons why this indicator of food-related material deprivation is the only systematically collected data regarding Europeans’ food access. First, the assumption that Europe does not have food access issues, and second, the fact that food insecurity has not been studied as an independent phenomenon, but as a component of other social issues, such as poverty or agricultural policy [29].

The only other Europe-wide data available come from the FAO State of Food Insecurity Report. FAO employs the Food Insecurity Experience Scale (FIES), which measures individual food insecurity and allows cross-country comparisons [43]. This tool is comparable to other validated experience scales [53], such as the Household Food Insecurity Access Scale (HFIAS) [62], or the USDA Household Food Security Survey Module (HFSSM) [42] that measure household food insecurity and are frequently used for research studies in specific population groups across Europe [[63], [64], [65]]. These instruments evaluate self-reported food-related behaviors and experiences concerning difficulties in accessing food due to limited resources. They focus on the economic aspect of food insecurity and provide a score indicating the degree of food security or insecurity. These levels include food security (guaranteed food access), marginal or low food insecurity (no economic access issues, but concerns), moderate food insecurity (difficulties accessing quality food), and severe food insecurity (insufficient food quantity). Note that the marginal or low level, despite not requiring a change in dietary intake, has significant health implications and should be reported separately from the food security level.

In the absence of routine food insecurity monitoring, the utility of proxy indicators to longitudinally determine the prevalence of food insecurity [66] has been examined. Food bank utilization has been the only available indicator of food insecurity to characterize and quantify it for many years. However, it does not represent the food-insecure population, as only a portion of food-insecure households request food assistance, and significant differences exist between the 2 groups [67,68].

Price and affordability are crucial factors contributing to food and nutrition insecurity, which poses significant barriers to accessing sufficient, safe, nutritious food to meet dietary needs, and food preferences for an active and healthy life [69]. Therefore, affordability is increasingly used as an indicator of food and nutritional inequality [70,71]. It is measured by determining the least-expensive energy-adequate, nutrient-adequate, or FBDG-compliant diet that is priced using either in-store at the different countries or retail data from the World Bank’s International Comparison Program.

Other proxy measures, such as household income, income-related poverty, changes in social security affiliations, household costs, food costs, or trends in malnutrition and health markers [66], have also been suggested as potential indicators of food insecurity. These can provide information on the causes of food and nutrition insecurity among a specific population and assist in identifying those at risk. Ultimately, however, even though they are related, proxy indicators are insufficient; standardized measures that go beyond traditional measurement scales and incorporate nutritional and social sufficiency are needed [66].

In Europe, the assessment of nutrition security is less developed than that of food security and is rarely integrated with assessments of food insecurity. Indicators include measures of dietary diversity (Household Dietary Diversity Scale, Diet Quality Questionnaire) and nutritional status, with the prevalence of undernourishment being the most common global measure [72]. The Committee of Food Security’s definition of nutrition security includes sanitary environments, safe water, adequate health services, and proper care and feeding practices as prerequisites for nutritional adequacy and nutritional status [9]. However, these measures do not adequately capture information on these prerequisites.

The most recent contribution to the measurement of food insecurity is the 2022 report from the FAO’s High Level Panel of Experts (HLPE), which requested the refinement of data collection and analysis tools in order to obtain timely and sufficiently granular data on food access, food intake, and nutritional status [73]. The report emphasized the need to develop measures that incorporate nutritional and dietary intake, diet quality, qualitative data on the psychosocial impact of food insecurity, and relevant individual, household, and social conditions associated with food insecurity, including food-system approaches.

There is a lack of systematically collected information on all aspects of the access dimension of food security and a general absence of data about the utilization dimension of food insecurity, which links the structural constraints for food security and the health (and social) effects of food insecurity. Monitoring food insecurity is not only important for addressing the phenomenon, but it can also be used to identify populations at increased risk of poor health in order to provide targeted interventions [74].

Several EU-funded projects have addressed the issue of food insecurity and developed frameworks for identifying elements to monitor and act upon [[75], [76], [77]], focusing on national food security through the lenses of the agrifood system which considers structural determinants like food production and supply aspects. They do not, however, address food insecurity at the household and individual levels, nor their main drivers in high-income countries, where food supply is relatively assured despite the fact that climate change and civil conflict pose ongoing threats to food availability.

### What is known about the prevalence, correlates, and coping strategies of food and nutrition insecurity in Europe?

In recent years, primary research on food insecurity in Europe has increased. Since 2010, the number of publications with the term “food insecurity” in the title or abstract that refer to European countries has steadily increased, from <10 publications per year until 2015 to 51 publications in 2022. Before 2010, only 3 articles were identified in PubMed. These articles examine food security in European countries during the 1900s and early 2000s, and report data from transitioning economies, including the Russian Federation [78], the Baltic Republics [79], and Kosovo [80]. In the context of more developed economies at the time, research on food insecurity focused on “externally” vulnerable populations, such as refugees [81]. After the 2008 economic crisis, which resulted in increased poverty rates and precaritization of employment—particularly in southern European countries [82], the scholarship on food insecurity in the European countries began to develop in 2010, despite some data published from before the crisis [[63], [64], [65]]. Regarding the number of indexed publications, 2 significant events can be identified: the 2008 economic crisis and the 2020 COVID pandemic lockdowns. However, research outputs do not necessarily reflect the countries most affected by the 2008 crisis, with the majority of publications coming from both severely affected (Portugal and Greece) [83] and less affected (France, the Netherlands, and the United Kingdom) [83].

Most studies on food and nutrition insecurity in Europe have sought to provide data about the prevalence and correlates of food insecurity in various populations [1,63,84]. However, some studies have discussed the personal experiences and coping strategies of food-insecure populations [[85], [86], [87]], describing aspects of food aid organizations as the primary response in most countries [88], or identifying food-system issues such as food redistribution [89] or trade dependency [8].

#### Prevalence

According to the food-related material deprivation indicator in the, in 2021, 7.3% of the total population and 17.4% of those at risk of poverty were unable to afford a meal containing meat, fish, or a vegetarian equivalent every other day [60]. At the national level, the percentages ranged from <1% in Cyprus; <5% in Estonia, Finland, Denmark, Sweden, Portugal, Luxembourg, Netherlands, Spain or Ireland; to >10% in Germany, Romania, Slovakia, Greece, and Hungary, reaching ∼22,5% in Bulgaria. Figures in the different countries more than double for at risk of poverty population. Data from nationally representative surveys or primary studies in selected samples consistently show an overall prevalence ranging from 8.4% to 10.0% (Denmark 2015; France 2011; EU 2015) [71,90,91] to ∼25% (United Kingdom 2007, Belgium 2020) [65,92]. In addition to the country and year of data collection, differences in prevalence rates can be attributed to the sampling strategies and methods employed to estimate food and nutrition insecurity. The majority of studies utilized variations of the USDA HFSSM survey [1,[63], [64], [65],^90^,^91^,[93], [94], [95]], but the FIES scale and HFIAS were also used [[96], [97], [98], [99]]. Several studies utilized the Eurostat and European Quality of Life Survey food deprivation item data [71,100,101]. These surveys, along with others conducted in the United Kingdom, Portugal, and Denmark, are based on representative samples [90,94,95,98].

Also proliferating in Europe are studies on the affordability of nutritionally adequate diets [69,71,[102], [103], [104], [105], [106]]. These studies, which indicate that from 0.3%–3.5% to ∼10% of the population cannot afford a healthy diet, contribute to the evidence that a significant portion of the European population is experiencing financial constraints that impact their ability to eat a healthy diet. The difference in magnitude is explained by the methodologies employed (references and methodological approach to construct the food basket for a healthy diet and in-store compared with survey-based pricing), the reference population (general compared with subpopulations), and the geographic area used as a reference (continental Europe compared with European Union). These are indications that the current social protection mechanisms are failing to protect the right to adequate food and nutrition, and that more generous and effective welfare systems may be able to mitigate the effects of recent crises [107]. Data from Greece [108], Germany [99,109], United Kingdom [110], and the Netherlands [111] show that over 70% of food aid recipients are food insecure, demonstrating that current protection measures do not adequately address food and nutrition insecurity [100]. Chatzivagia et al. [108], for example, compared the health status and nutritional intake of the Greek population receiving food aid from the Fund for European Aid to the Most Deprived (FEAD) in terms of nutrition insecurity. In comparison to the general population, FEAD recipients had lower intakes of energy, fat, and monounsaturated fat, and higher intakes of carbohydrate, total protein, protein from plant sources, and fiber, and despite receiving food aid, they were more likely to have a poor diet and be malnourished.

However, notwithstanding the methodological approaches, the data from these primary studies are insufficient to provide a systematic overview of the evolution of food insecurity in Europe. Moreover, despite the increase in research outputs concerning these economic and social shocks, they are unable to accurately assess the impact of the 2008 and COVID crises. Although some studies reported an increase in the prevalence of food insecurity (for example, [92,112,113]), it is unclear whether this impact has been sustained.

#### Correlates

Beyond data on the rate of food insecurity in different groups, several studies have examined risk factors and correlations for food insecurity (for example, [63,65,94,96,97,100,101,[114], [115], [116]]), revealing its association with structural conditions such as poverty, unemployment or low work intensity or employment precarity, housing tenure, low incomes, and low levels of education. The increase in food insecurity risk in households headed by women and those with children is well-documented [[117], [118], [119], [120]], and new research is emerging on its prevalence among youth [96,116] and older adults [97,121].

#### Experiences and coping strategies

Several studies have investigated the personal experiences, resources, and obstacles faced by populations with food insecurity. Gracia-Arnaiz [86] investigated the food practices of 51 first-time applicants for social assistance in Spain. They found that participants employed a variety of strategies, such as altering the quality of foods (with a focus on satiety rather than nutrient density), shopping less frequently and/or at different stores, seeking out cheaper brands, preparing simpler dishes, growing food, and recycling leftovers. Participants’ itineraries showed an increasing reliance on the charitable sector for food and the use of other community resources, such as family, neighbors, and activist organizations. Multiple qualitative studies corroborated these findings, demonstrating food insecurity’s nutritional, physical, and psychological effects. Concerns about sustainability, stigma, and shame are being raised regarding the reliance on charitable food aid as the primary response to food insecurity. A more affordable food supply for everyone would be more desirable than food aid for the “deserving poor” to achieve a healthy and adequate diet [85,87,122].

### Responses to food insecurity in Europe

The mechanisms for addressing food insecurity in Europe include a combination of European and national welfare policy measures and food assistance programs.

#### Welfare policies

National welfare policies and social assistance measures are the region’s primary instrument for social protection. These include unemployment, housing/social exclusion, illness-health care, disability, retirement age, and family/child benefits. Increased access to income aids in the prevention and increase of food and nutrition insecurity by addressing its structural causes. In 2020, €4074 billion [30.4% of the global gross domestic product (GDP)] was spent on social protection benefits in Europe, with the largest share of the expenditure going to the old age and survivors (mainly consisting of pensions provided to elderly individuals and surviving family members to ensure a decent standard of living in their later years) (44.2%), followed by healthcare and sickness pensions that include the coverage for medical expenses and income replacement during periods of illness or disability (29.1%) [123]. Altogether, the protection benefits for family/children (for example, child allowances, parental leave benefits, and childcare subsidies), unemployment (for example, financial aid provided to unemployed individuals and actively seeking work), and housing/social exclusion (for example, various support programs aimed at preventing homelessness, promoting affordable housing, and addressing social exclusion) represented <20%. Significant differences exist between EU Member States in terms of both the types of social benefits offered and their proportion of GDP. In 2020, France, Italy, and Austria showed the most on social benefits (over 33%), whereas Ireland, Romania, and Latvia invested <18% [123]. Eurostat data also revealed substantial disparities in the proportion of national budgets allocated to specific social benefits based on demographic, socioeconomic, and political circumstances and priorities. Consequently, each member state determines the eligibility requirements for its citizens and households to receive social benefits. In the average case, >65% of these benefits are cash transfers [123]. There is evidence that social protection programs can mitigate the effects of unemployment and declining wages on food insecurity [1,100,105,124], but they are insufficient as a stand-alone measure [102].

Additionally, some countries may implement fiscal and regulatory measures at the food-system level. These include reduced taxes on certain basic products and staples [125] and subsidies for the producers of specific foods [126].

#### The FEAD program

Since 2014, the European Commission‘s primary response to food insecurity has been the FEAD, the successor to the European Food Aid Program for the Most Deprived Persons, which was in operation from 1987 until the introduction of FEAD in 2014. The FEAD was originally designed for 2014–2020, but it was extended to 2022 due to the COVID-19 pandemic [127]. Starting in 2023, the FEAD program will be incorporated into the European Social Fund + (ESF+) 2021–2027.

The FEAD (and, beginning in 2023, the EFS+) supports European countries’ efforts to provide food and/or basic material assistance to the most disadvantaged, to assist households and individuals in taking their first steps out of poverty. For 2014–2020, over €3.8 billion was allocated for the FEAD, which was later supplemented with additional funds for 2020–2022. European countries must contribute at least 15% of the funds they receive to co-finance their national program, and food aid is contingent on socioeducational, labor, and other supports. Each member state is free to manage the funds as they see fit. In countries such as Belgium, France, and Spain, governments use FEAD funds to make open bids to which the food industry can respond with surpluses or increased production. Others, such as Cyprus or Hungary, co-finance initiatives to improve the nutritional status of the poorest, such as school breakfast programs. Not all countries use FEAD funds to purchase food. In the Netherlands, for instance, the funds are used to reduce the social exclusion of low-income senior citizens [128].

In 2019, over 12.2 million people received food assistance; between 2014 and 2018, over 1.6 million tons of food was distributed. This assistance is supplemented by measures to promote the social inclusion of final recipients, such as referring them to appropriate services, providing advice on a balanced diet, and offering assistance with household budgeting. Member States work with partner organizations (public bodies and nonprofits) to implement FEAD programs and distribute food aid to recipients.

In contrast to traditional forms of food aid, which rely on surplus food or donations, FEAD relies solely on purchased products. In this way, unlike charitable organizations that rely on donated or rescued food, FEAD ensures a steady and consistent supply of products by not relying on donations. However, its effectiveness to alleviate poverty remains unknown, and it is contested in terms of food-system equity, respect for human dignity, and how it contributes to promoting an adequate nutritional intake [129,130].

The ESF+ program will operate differently than FEAD, with voucher cards taking precedence over in-kind produce, among other modifications. Unknown are the specifics of how each country will implement the new ESF+ program, but the possibility of providing support through vouchers and monetary cards is largely viewed as a step in the right direction, given the opportunities in terms of agency, social equity, and nutritional adequacy [131].

#### Food assistance programs

After social protection benefits and the FEAD program, the third line of defense against food insecurity is the development of government and nongovernment organizations’ food assistance programs. These include traditional resources such as food banks and soup kitchens and more unconventional and innovative proposals such as voucher cards, social gardens, food service redistribution, and community kitchens. Collaborative initiatives such as neighborhood networks, fiscal measures, initiatives to promote access to fresh food for vulnerable populations, and food recovery programs, among others, also contribute to reducing access disparities and fostering nutritional stability [132]. In addition, most countries employ specific measures for particular population groups. In particular, these actions recognize the specific needs of children and older people, in the form of subsidized school meals, community centers, or meals at home [87,133,134]. Although cultural preferences are beginning to be considered in the distribution of food aid, nutritional and disease-related dietary requirements are rarely observed [135,136].

Substantial evidence suggests that these strategies are insufficient for achieving food security and ensuring that users’ nutritional needs are met [108,133,137]. Originally intended for emergencies, food assistance devices such as food banks are now used as a long-term solution for food insecurity. While the new approaches (voucher cards, social gardens, food recovery programs, and community kitchens) have the benefit of promoting a more adequate access to food and social bonding [138,139], they remain “band-aid” solutions that do not address the upstream determinants of food insecurity and can divert attention and resources from achieving long-term food security (for example, [130,139,140]).

This difficulty in adequately addressing food insecurity in European countries is rooted in how it is conceptualized politically. Article 25 of the UN Declaration of Human Rights recognizes the right to food. Although the majority of European countries have ratified the declaration, the right to food is not mentioned in the constitutions of any of the member states. In Latin America, for instance, Bolivia, Brazil, Cuba, and Mexico have incorporated the right to food into their respective constitutions. Certain countries have also signed on to the FAO’s Voluntary Guidelines to support the progressive realization of the right to adequate food within the context of national food security, but these guidelines must be operationalized in the various European territories. As a result, there are insufficient political instruments to ensure that everyone has access to adequate food and nutrition. In contrast, a patchwork of responses is offered, with a heavy reliance on the nongovernment, not-for-profit, and charitable sectors to fill the void, indicating market failure. As previously described, hunger and food insecurity are distinct entities, and the right to adequate food and nutrition is distinct from the right not to experience hunger [141]. Food security includes access to safe and nutritious food [34], so it is necessary to provide not only edible products that provide sufficient energy, but also foods that meet all the nutritional requirements for an active and healthy lifestyle.

Responses to food insecurity must be articulated through policy instruments in a proactive, as opposed to reactive, manner, and must incorporate both population and high-risk strategies. Food and nutrition security is a complex, cross-systems issue that requires the participation of a wide range of stakeholders across the food system and other political domains [32,142]. Dietitians are ideally positioned to offer guidance and expertise [143].

### Challenges and new directions

Achieving food and nutrition security in Europe requires deploying the means to guarantee that everyone, regardless of their circumstances, has regular access to sufficient, safe, and nutritious food to maintain a healthy and active lifestyle, and that food is accessed in socially acceptable ways. These measures require the existence of monitoring and screening systems, the addressing of structural conditions that threaten food insecurity, such as unemployment, underemployment, low wages, and the affordability of healthy diets, and a food safety net that enables dignified and socially acceptable access to food in the short term. In addition, they include the actions required to ensure that those experiencing or at risk of food insecurity, as well as the general population, have adequate food literacy to make the most of their food. Food-insecure individuals should also be guaranteed the right to nutritional care [144]. This objective can only be achieved through systemic transdisciplinary actions based on a solid food and nutrition security understanding. Dietitians are ideally suited to contribute to addressing food and nutrition insecurity in Europe due to the significant dietary and health consequences associated with food and nutrition insecurity and their primary focus on food as food-focused health professionals.

Although data regarding the current contributions of dietitians to food and nutrition insecurity in Europe are unavailable, the fact that institutions such as the Red Cross, Caritas, and other nongovernment and not-for-profit organizations are incorporating dietitians into their teams, more local or regional governments rely on dietitians to develop and/or implement new approaches toward healthy and sustainable food for all, and the proliferation of university programs in dietetics suggest that dietitians play a significant role in addressing food and nutrition insecurity. However, this potentially invaluable role for dietitians is still in its infancy. There is still a significant portion of the dietetic profession that is unaware of the levels of food and nutrition insecurity in the region and the impact that these insecurities can have on short- and long-term health.

The dietitian’s first responsibility is to raise awareness, both within and outside the profession, that food and nutrition insecurity in Europe is a persistent symptom of social and health inequality. Not only is the lack of timely data on the status of food insecurity in Europe alarming [29], but the previous HLPE report also highlighted the difficulty of making relevant information available and accessible to policymakers to inform decision making and inspire action [73]. Dietitians are in a privileged position to disseminate information about food and nutrition insecurity to other stakeholders and the general public, highlighting how it threatens dietary quality and is a risk factor for overall health and well-being, child development, and numerous diet-related diseases and conditions. It may be important to raise awareness within the health sector to identify obstacles to health promotion, disease prevention, and treatment. Dietitians in academia or those involved in the education of future dietitians (for example, in vocational training or internships) are tasked with ensuring that the next generation of professionals is aware of food and nutrition insecurity, its consequences, and how to address it. They must ensure that the profession is aware of the structural causes of food insecurity and to advocate for political change with courage.

Dietitians work with individuals regarding their diets and eating habits; as a result, dietitians can actively screen for food insecurity in hospitals, primary-care centers, and community-care facilities, enabling the identification of food-insecure individuals and households. Dietitians undertake a social assessment as part of the nutrition care process and are particularly vigilant to risk factors for food insecurity such as gender, age, migration status, household type, educational level, occupation status, and health insurance. As such, they can adjust their advice and link to appropriate food safety nets to ensure adequate dietary intakes across the lifespan.

Dietitians in Europe are well-positioned to contribute to the production of knowledge through research that helps to characterize food insecurity in the region, given their research capacity. Food and nutrition insecurity must be monitored at the national and regional levels to determine its prevalence, underlying causes, and associated factors. Dietitians play a crucial role in selecting, developing, and enhancing appropriate and reliable methods for estimating food insecurity, and in guiding the collection of dietary and nutritional data. Dietitians can also contribute by collaborating with sociologists, economists, and policy experts to design and evaluate the efficacy and viability of social benefits and food assistance programs.

Dietitians have the capacity to collaborate with various stakeholders in developing policy instruments and interventions that facilitate the availability and accessibility of adequate food and nutrition. This includes informing welfare policies that guarantee food security for all and foster healthier and sustainable food systems. In addition, dietitians play a crucial role in the development of food aid systems that consider diet quality and the social functions of food, while reducing the stigma for participants. Practically, dietitians contribute to knowledge sharing by educating others on screening processes and evidence-based interventions, the nutritional value of food aid packages, and the nutritional status and health risks of food-insecure populations, including food aid users.

Dietitians are therefore part of the solution and must accept the challenge of recognizing food and nutrition insecurity as a significant and real problem in Europe. They must contribute their voice and practical abilities and collaborate with a variety of stakeholders with complementary expertise to develop transdisciplinary, comprehensive, intersectoral, and integrated strategies to ensure that all Europeans can realize their right to food, leaving no one behind.

## Acknowledgments

ECA is grateful to Danielle Gallegos and Amanda Avery, for their valuable feedback and insightful suggestions, which greatly contributed to the improvement of this manuscript.

### Author contributions

The author’s responsibilities were as follows—ECA: responsible for design, writing, and final content of the manuscript.

### Conflict of interest

The author reports no conflicts of interest.

### Funding

No funding was received for conducting this study.

## Footnotes

1 Eastern Europe: Belarus, Bulgaria, Czechia, Hungary, Poland, Republic of Moldova, Romania, Russian Federation, Slovakia, and Ukraine; Northern Europe: Denmark, Estonia, Finland, Iceland, Ireland, Latvia, Lithuania, Norway, Sweden, and United Kingdom of Great Britain and Northern Ireland; Southern Europe: Albania, Andorra, Bosnia and Herzegovina, Croatia, Greece, Italy, Malta, Montenegro, North Macedonia, Portugal, Serbia, Slovenia, and Spain; Western Europe: Austria, Belgium, France, Germany, Luxembourg, Netherlands, and Switzerland.
